# A Case of Valproate-Induced Hyperammonemic Encephalopathy

**DOI:** 10.7759/cureus.9114

**Published:** 2020-07-10

**Authors:** Faraaz Zafar, Beau M Billadeau, Ahsen U Ahmed

**Affiliations:** 1 Internal Medicine, Gundersen Health System, La Crosse, USA

**Keywords:** toxic encephalopathy, side effects of medical treatment, drug-related side effects and adverse reactions, neurologic complications

## Abstract

A 56-year-old Caucasian male with a history of seizure disorder on long-term prophylaxis with valproate presented with altered mental status, aggressive behavior, decreased oral intake, and frequent myoclonic jerking movements. Electrolyte and other basic metabolic lab testing, liver function testing, and imaging studies were negative for acute abnormalities or infection, though ammonia levels returned markedly elevated, and he also had a macrocytic anemia despite having normal folate and B12 levels. Following discussions with neurology, his valproate was felt to be the inducing factor for his hyperammonemic encephalopathy. After discontinuation of valproate and changing to a new anti-seizure medication, he soon returned to his neurologic baseline. This case report evaluates his presentation and current literature on hyperammonemic encephalopathy induced by valproate.

## Introduction

Valproate is a medication often used in the treatment of epilepsy, bipolar disorder, and prevention of migraines. However, its exact mechanism is currently not well understood. Encephalopathy due to hyperammonemia is a well-established and serious adverse reaction to valproate. While several cases exist regarding the relationship between valproate and hyperammonemic encephalopathy, the exact mechanism of action remains to be elucidated. Furthermore, there are no well-established treatment guidelines for cases when this does occur. This case report gives another example of this recognized problem and the management options pursued.

## Case presentation

A 56-year-old Caucasian male from a group home, who was nonverbal at baseline with a history of cerebral palsy, spastic quadriplegia, seizure disorder on valproate for two years prior to current presentation, hyperlipidemia, and aspiration, presented to the emergency department (ED) with his caregiver. His visit was due to increasing agitation, aggressive behavior, crying out, refusing to eat, and notable frequent myoclonic jerking movements lasting five to ten seconds. He was not postictal following these myoclonic movements. He remained afebrile and was hemodynamically stable. ED work-up was significant for negative chest X-ray, negative kidney, ureter, and bladder (KUB), and normal electrolytes, creatinine, and lactate. However, ammonia levels returned elevated at 240 µmol/L. Liver function testing showed no abnormalities with total bilirubin 0.3 mg/dL, and urinalysis was negative for evidence of infection. His complete blood count revealed a normal white count, though he had a macrocytic anemia with hemoglobin of 13.5 g/dL and mean corpuscular volume (MCV) of 106.6 fL despite folate and B12 being within normal limits. Valproate was found to be within therapeutic range at 91 µg/mL. Per brief review of the literature, it was determined that valproate can affect the urea cycle, leading to a buildup of ammonia. Therefore, there was concern that valproate was the inciting cause of this patient’s presentation. After discussions with the neurology service, recommendations were made to discontinue his valproate, start levetiracetam, and treat his hyperammonemia with lactulose (30 g three times daily) and L-carnitine (starting at 50 mg/kg bolus with 25 mg/kg every eight hours until ammonia levels decreased). The patient was admitted for observation with the aforementioned cares.

The following day he was noted to be less agitated and having less frequent myoclonic jerking, with lab testing showing a decrease in ammonia levels from 240 to 116 µmol/L, and he was felt by his group home caregivers to be around his baseline. His ammonia levels had decreased to 62 µmol/L, and he had no further episodes of agitation or myoclonic jerking movements. He was maintaining appropriate oral intake. The L-carnitine and lactulose were discontinued, and the patient was subsequently discharged to a group home with a regimen of levetiracetam 500 mg BID, as was started during the hospitalization.

One week after discharge, he was readmitted for seizure-like activity, and was noted during his stay to have a generalized seizure with eye deviation, drooling, and rhythmic jerking motion of upper extremities. Lab testing was unremarkable at the time (including normal ammonia and valproate levels), and it was felt that his seizures were due to inadequate medication dosage. He received IV levetiracetam and his oral dose of levetiracetam was increased to 750 mg BID. He had no further episodes of seizure activity and was discharged back to the group home the next day. At the last evaluation six months after presentation, he had been stable without seizures since that time.

## Discussion

Valproate is a branched short-chain fatty acid (SCFA) used for a variety of medical conditions, most commonly for seizure prevention, migraine prevention, or treatment of bipolar disorder. Hyperammonemia induced by valproate has been reported numerous times, as early as 1993 [1]. Recent evidence suggests that this may not be a rare occurrence and may occur in a significant number of patients on valproate. A recent retrospective study was performed of 347 patients who were admitted to the psychiatric unit of a community teaching hospital, and had received at least one dose of valproate. Analysis found hyperammonemia (defined as ammonia > 47 μmol/L) in 36% of patients. Of these, 43.2% of patients were symptomatic and valproate discontinuation was found to be the most effective treatment (56.3% success rate) [2]. However, this study was limited by the lack of criteria for defining symptomatic hyperammonemia. Another prospective, cross-sectional study of 107 patients on valproate for mood disorders or epilepsy found valproate-induced hyperammonemia (VIH) (defined as ammonia > 52 μmol/L) in 55.3% of cases with a dose-dependent association between valproate and blood ammonia level [3].

**Figure 1:**
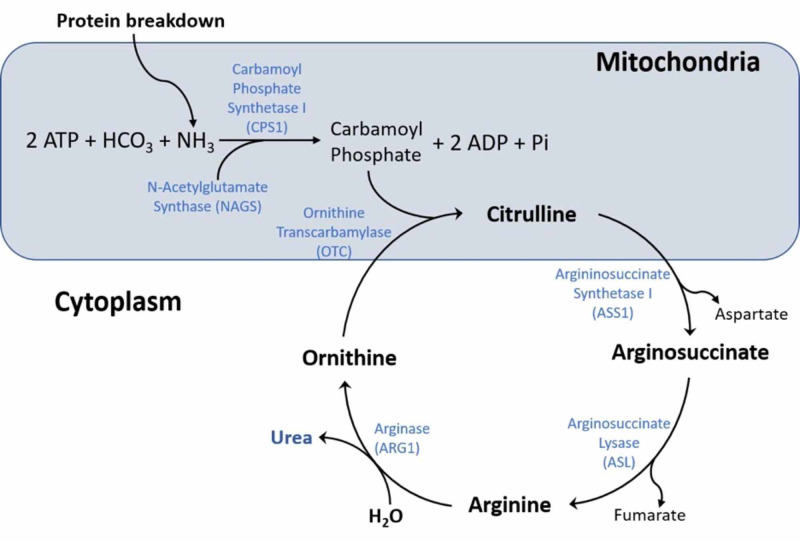
The urea cycle in hepatocytes converts ammonia to urea through a series of steps. It is believed to be impaired in patients with valproate-induced hyperammonemia, although the exact etiology remains unclear.

The mechanism of action of valproate is not fully understood, with proposed mechanisms including blocking voltage-gated sodium channels, blocking T-type calcium channels, increasing levels of gamma-aminobutyric acid (GABA) in the brain with subsequent GABAergic effect, and through inhibition of histone deacetylases [4]. The exact molecular pathway by which hyperammonemia occurs in the setting of valproate use has not yet been fully elucidated. It is known that valproate is metabolized through the liver through a mixture of glucuronide conjugation and mitochondrial beta oxidation [4]. The liver is responsible for the breakdown of ammonia, which begins in the hepatocyte mitochondria and concludes in the cytoplasm with the production of urea. The initial step of the urea cycle (Figure 1), also the rate-limiting step, is the conversion of ammonia and bicarbonate into carbamoyl phosphate using the carbamoyl phosphate synthetase I enzyme (CPS I) [5].

Multiple research studies have suggested the inhibition of CPS I by valproate to be the cause of hyperammonemia. Recent research has shown that the CoA ester of valproate, produced from the metabolism of valproate, inhibits the production of citrulline in the mitochondria via inhibition of N-acetylglutamate synthase (NAGS). This was confirmed with the addition of N-acetylglutamate substrate and the increased activity of NAGS leading to increased citrulline production. This makes the prior thoughts of valproate directly inhibiting CPS I less likely [5,6]. Previous studies and case reports have noted that a possible inciting factor may be genetic mutations in the CPS I and the ornithine transcarbamylase (OTC) enzymes that are critical for urea cycle offering an explanation for VIH only occurring in a subset of patients [7,8]. Studies do show, however, that many patients with hyperammonemia may remain asymptomatic, and as such may not require any treatment, and ammonia levels may not necessarily correlate with severity of encephalopathy [9].

Of note, it is interesting that variation exists, with some studies showing lab testing with high to high-normal valproate levels, and others indicating normal valproate levels, such as in this case [7,8]. Furthermore, it should be pointed out that in this case, the patient had been on valproate for two years prior to this complication arising from therapy. This compares with other studies, where patients had been on valproate therapy for between one to three months prior to complications of hyperammonemia arising [1,7,8]. Later onset is recognized, but appears to be less common [9]. This may serve as a further warning to providers to remain vigilant for an extended period of time following initiation of valproate therapy.

## Conclusions

VIH is an uncommon but serious side effect of valproate in a subset of patients. Thus, it should be investigated as a possible cause of encephalopathy in patients on valproate in cases of both acute and chronic use. Elevation of ammonia level from baseline in such cases is especially concerning for VIH in the absence of a more plausible alternate explanation and should prompt treatment for suspected valproate-induced hyperammonemic encephalopathy (VHE). In the case presented here, cessation of valproate, and short-term administration of L-carnitine and lactulose, resulted in durable resolution of the patient’s symptoms. The exact etiology of VIH and VHE remains unclear, but it is believed to be due to impairment of the urea cycle due to valproate in susceptible patients. Currently, there are no definitive recommendations on monitoring valproate or ammonia levels in patients who take the medication. Given the fact that valproate levels may be normal in many cases, this may be left to provider discretion. However, monitoring ammonia levels should certainly be considered. We hope that this case can help to serve as a reminder and warning to clinicians that long-term development of valproate complications may still occur, and to remain vigilant and consider periodic monitoring of ammonia levels.
